# Supplementary material for: Local adaptation and future climate vulnerability in a wild rodent
Source: Nat Commun. 2023 Nov 29;14:7840. doi: 10.1038/s41467-023-43383-z (PMC10686993; doi:10.1038/s41467-023-43383-z)
Supplement: Supplementary file 1 — Supplementary Information [file 41467_2023_43383_MOESM1_ESM.pdf]

Supplementary Information

Local adaptation and future climate vulnerability in a wild rodent

Marková et al.

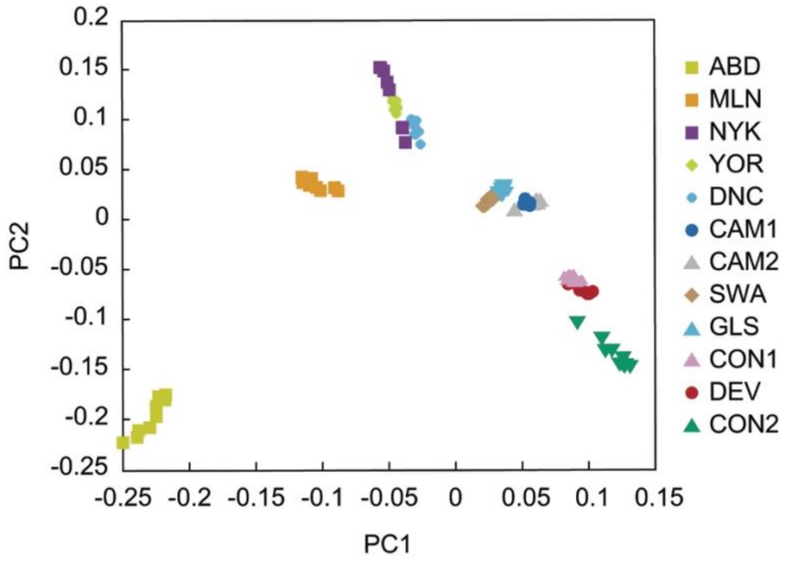

Supplementary Fig. 1. Principal component (PC) analysis of bank vole populations in Britain. Population codes refer to Supplementary Table 1.

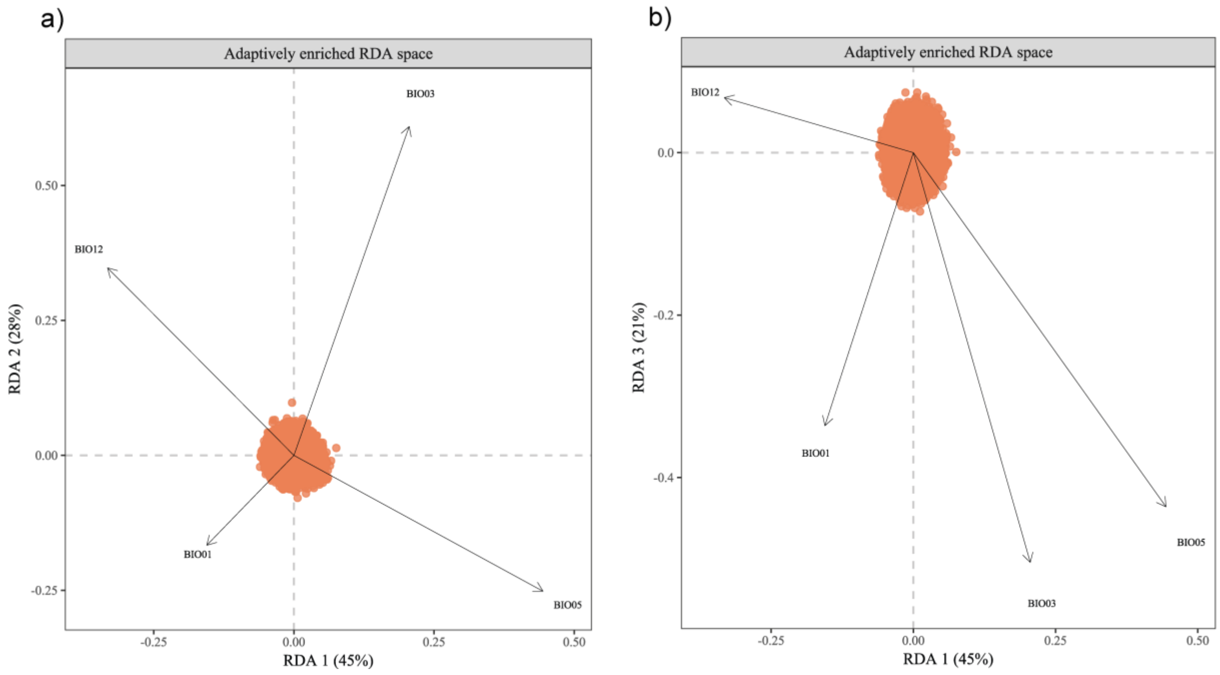

Supplementary Fig. 2. Adaptively enriched genetic space showing association between adaptive loci and climatic drivers of adaptation. Bioclimatic variables retained by the partial redundancy analysis (pRDA): BIO1 = annual mean temperature, BIO3 = Isothermality, BIO5 = max temperature of warmest month, BIO12 = annual precipitation

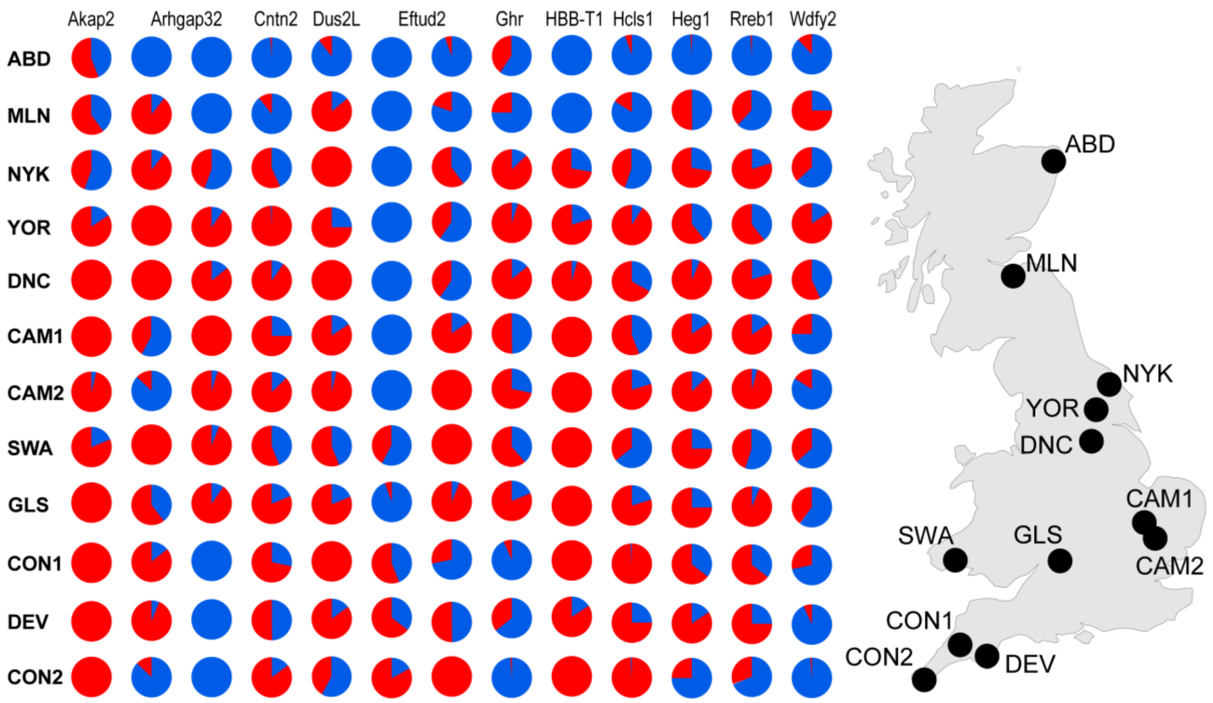

Supplementary Fig. 3. Frequencies of alternative alleles (blue and red) for candidate adaptive genes related to adaptation to temperature and humidity. Populations are arranged from north to south (top to bottom) and their codes refer to Supplementary Table 1. Map was created using ArcMap (v.10.8) and the Esri World Countries dataset (https://www.esri.com).

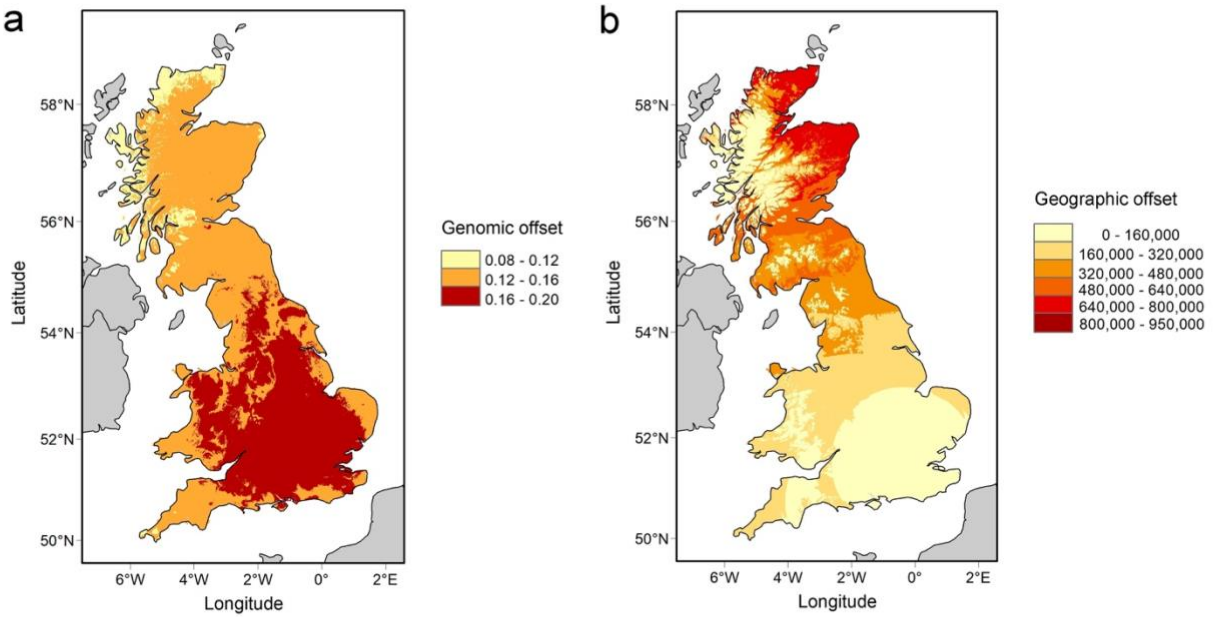

Supplementary Fig. 4. (a) Genomic offset and (b) geographic offset (in meters) predicted by the gradient forest approach. The maps were created using ArcMap (v.10.8) and the Esri World Countries dataset (https://www.esri.com).

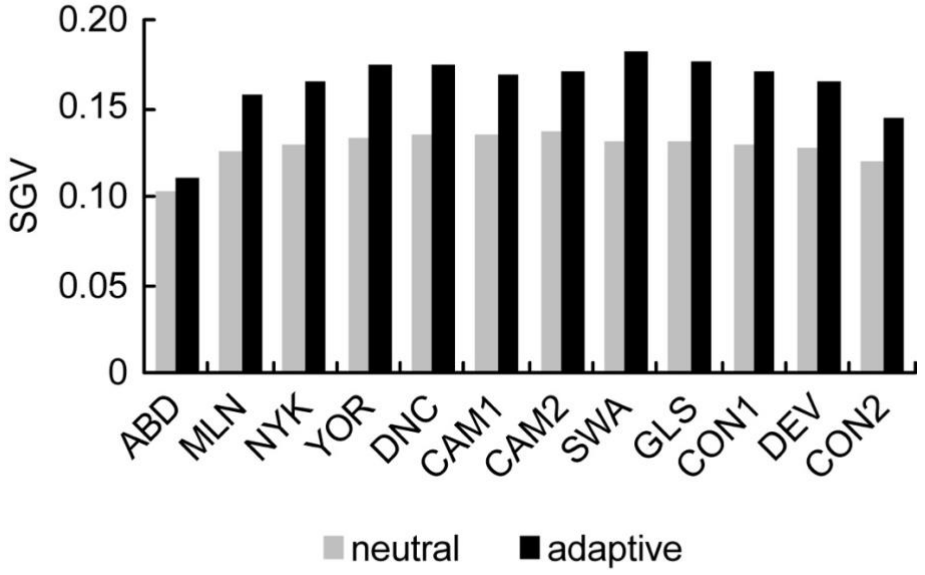

Supplementary Fig. 5. Standing genetic variation (SGV) in each population estimated for adaptive candidate SNPs and for comparison for putative neutral SNPs, i.e., SNPs not identified as outliers by any method. Population codes refer to Supplementary Table 1.

Supplementary Table 1. The origin of bank vole population samples

Code	Locality	County	Lat.	Long.	N
ABD	Maud	Aberdeenshire	57.48	-2.08	9
MLN	Roslin	Midlothian	55.85	-3.10	10
NYK	Dalby Forest	Yorkshire	54.29	-0.68	9
YOR	Heslington	Yorkshire	53.93	-1.01	10
DNC	Doncaster	Yorkshire	53.49	-1.13	10
CAM1	Wicken	Cambridgeshire	52.28	0.27	6
CAM2	Temple End	Cambridgeshire	52.13	0.41	12
GLS	Cirencester	Gloucestershire	51.77	-1.89	10
SWA	Tavernspite	South Wales	51.78	-4.64	8
CON1	Trebartha	Cornwall	50.57	-4.44	7
DEV	Diptford	Devon	50.41	-3.76	10
CON2	Lizard Peninsula	Cornwall	50.05	-5.18	10

Supplementary Table 2. Pairwise F_{st} values between bank vole populations.

Pop*	**ABD**	**MLN**	**NYK**	**YOR**	**DNC**	**CAM1**	**CAM2**	**GLS**	**SWA**	**CON1**	**DEV**
**ABD**											
**MLN**	0.139										
**NYK**	0.187	0.076									
**YOR**	0.177	0.066	0.042								
**DNC**	0.175	0.065	0.054	0.043							
**CAM1**	0.195	0.096	0.079	0.068	0.057						
**CAM2**	0.204	0.105	0.089	0.078	0.066	0.015					
**GLS**	0.193	0.090	0.074	0.063	0.052	0.027	0.036				
**SWA**	0.179	0.076	0.065	0.055	0.044	0.034	0.043	0.031			
**CON1**	0.217	0.119	0.105	0.096	0.084	0.049	0.057	0.056	0.046		
**DEV**	0.226	0.129	0.114	0.104	0.094	0.058	0.066	0.065	0.056	0.035	
**CON2**	0.260	0.165	0.152	0.142	0.131	0.096	0.104	0.105	0.094	0.072	0.081

* Population codes refer to Supplementary Table 1.

Supplementary Table 3. Number of outliers obtained with three different approaches.

Approach	No. of SNPs	No. of SNPs within know genes*	No. of SNPs within annotated known genes	No. of SNPs within HRG+	No. of non-synonymous SNPs	No. of annotated non-synonymous SNPs
**GO analyses‡**						
Fst 95th quantile	12,055	1,850	1,389	178	149	88
pcadapt FDR 10%	13,387	1,934	1,416	168	145	84
pRDA FDR 10%	20,383	2,832	2,102	235	190	103
intersection of *Fst*/*pcadapt*/pRDA	1,075	172	126	20	19	13
**Adaptive landscape**						
pRDA FDR 5%	13,910	1,991	1,471	164	134	74

* SNPs located within or less than 1,000 bp upstream or downstream of known genes in the bank vole genome
HRG+ hypoxia related genes
GO analyses‡ gene ontology analyses

Supplementary Table 4. The influence of climate (clim.), geography (geog.) and genetic ancestry (anc.) on genetic variation decomposed with partial redundancy analysis (pRDA).

**Partial models**	***df***	**Variance**	r^2	**Pr(>*F*)***	**Proportion of explainable variance**	**Proportion total of variance**
Total explainable: *F* ~ clim. + anc. + geog.	9	17,080	0.23	0.001	1.000	0.234
Pure climate: *F* ~ clim. | (anc. + geog.)	4	4,583	0.06	0.001	**0.268**	**0.063**
Pure geography: *F* ~ geog. | (clim. + anc.)	2	2,200	0.03	0.001	0.128	0.030
Pure ancestry: *F* ~ anc. | (clim. + geog.)	3	3,057	0.04	0.001	0.179	0.042
Confounded climate/geography/ancestry		7,240			0.423	0.099
Total unexplained		55,800				0.766
Total		72,880				1

* *p* value of the *F* statistic

Supplementary Table 5. The distribution of reference and alternate alleles in the twelve populations (see main text).

Population*	Percentage of fixed alleles	No. of fixed alleles
ABD	54.3	584
MLN	26.0	280
NYK	28.4	305
YOR	24.2	260
DNC	20.7	222
CAM1	30.7	330
CAM2	22.7	244
GLC	19.3	207
SWA	18.5	199
CON1	21.5	231
DEV	21.1	227
CON2	30.1	324

* Population codes found in Table S1
