# Supplementary material for: Local adaptation and future climate vulnerability in a wild rodent
Source: Nat Commun. 2023 Nov 29;14:7840. doi: 10.1038/s41467-023-43383-z (PMC10686993; doi:10.1038/s41467-023-43383-z)
Supplement: Supplementary file 3 — Description of Additional Supplementary Files [file 41467_2023_43383_MOESM3_ESM.pdf]

Description of Additional Supplementary Files

File Name: Supplementary Data 1

Description: Annotation of candidate loci
