# Supplementary material for: Local adaptation and future climate vulnerability in a wild rodent
Source: Nat Commun. 2023 Nov 29;14:7840. doi: 10.1038/s41467-023-43383-z (PMC10686993; doi:10.1038/s41467-023-43383-z)
Supplement: Supplementary file 5 — Reporting Summary [file 41467_2023_43383_MOESM5_ESM.pdf]

nature portfolio

Corresponding author(s): Petr Kotlík

Last updated by author(s): Oct 31, 2023

Reporting Summary

Nature Portfolio wishes to improve the reproducibility of the work that we publish. This form provides structure for consistency and transparency in reporting. For further information on Nature Portfolio policies, see our Editorial Policies and the Editorial Policy Checklist.

Statistics

For all statistical analyses, confirm that the following items are present in the figure legend, table legend, main text, or Methods section.

n/a	Confirmed	
☐	☒	The exact sample size (*n*) for each experimental group/condition, given as a discrete number and unit of measurement
☐	☒	A statement on whether measurements were taken from distinct samples or whether the same sample was measured repeatedly
☐	☒	The statistical test(s) used AND whether they are one- or two-sided<br>*Only common tests should be described solely by name; describe more complex techniques in the Methods section.*
☒	☐	A description of all covariates tested
☒	☐	A description of any assumptions or corrections, such as tests of normality and adjustment for multiple comparisons
☐	☒	A full description of the statistical parameters including central tendency (e.g. means) or other basic estimates (e.g. regression coefficient) AND variation (e.g. standard deviation) or associated estimates of uncertainty (e.g. confidence intervals)
☐	☒	For null hypothesis testing, the test statistic (e.g. *F*, *t*, *r*) with confidence intervals, effect sizes, degrees of freedom and *P* value noted<br>*Give P values as exact values whenever suitable.*
☒	☐	For Bayesian analysis, information on the choice of priors and Markov chain Monte Carlo settings
☒	☐	For hierarchical and complex designs, identification of the appropriate level for tests and full reporting of outcomes
☒	☐	Estimates of effect sizes (e.g. Cohen's *d*, Pearson's *r*), indicating how they were calculated

Our web collection on statistics for biologists contains articles on many of the points above.

Software and code

Policy information about availability of computer code

Data collection	Climate data are from a publicly available source cited in the manuscript. No software was used for data collection.
Data analysis	No custom software was used. We used the following third-party software for data analysis: Illumina bcl2fastq (v. 2.20.0.422), FastQC (v. 0.11.9), trimmomatic (v. 0.36), bwa (v. 0.7.10-r789), samtools (v. 1.6.0), picardtools (v. 2.18.5-6), bcftools (v. 1.13), vcftools (v. 0.1.17), RepeatMasker (v. 4.1.2), GenMap (v. 1.3.0), plink (v. 1.9), EIGENSOFT (v. 7.2.1), Admixture (v. 1.3.0), plink (v. 1.9), pcadapt (v. 4.3.3), R package qvalue (v. 2.15.0), R package vegan (v. 2.6.4), R package gradientforest (v. 0.1.34), CLC Genomics Workbench (v. 23.0.2), Panther (v. 17.0102), QuickGO (v. 2022-11-18), ggplot2 (v. 3.4.2) and ArcGIS (v.10.8).

For manuscripts utilizing custom algorithms or software that are central to the research but not yet described in published literature, software must be made available to editors and reviewers. We strongly encourage code deposition in a community repository (e.g. GitHub). See the Nature Portfolio guidelines for submitting code & software for further information.

Data

Policy information about availability of data

All manuscripts must include a data availability statement. This statement should provide the following information, where applicable:

- Accession codes, unique identifiers, or web links for publicly available datasets
- A description of any restrictions on data availability
- For clinical datasets or third party data, please ensure that the statement adheres to our policy

The raw Illumina sequencing data generated in this study are deposited at NCBI in the Sequence Read Archive (SRA) under BioProject PRJNA1017835 (https://www.ncbi.nlm.nih.gov/bioproject/PRJNA1017835) with accession numbers SRR26070875-SRR26070988. SNP genotyping data, reference genome assembly and annotation files are available in the Dryad Digital Repository (https://doi.org/doi:10.5061/dryad.kwh70rz96). WorldClim climate data are publicly available at (https://www.worldclim.org).

Research involving human participants, their data, or biological material

Policy information about studies with human participants or human data. See also policy information about sex, gender (identity/presentation), and sexual orientation and race, ethnicity and racism.

Reporting on sex and gender	This study does not include human research participants.
Reporting on race, ethnicity, or other socially relevant groupings	This study does not include human research participants.
Population characteristics	This study does not include human research participants.
Recruitment	This study does not include human research participants.
Ethics oversight	This study does not include human research participants.

Note that full information on the approval of the study protocol must also be provided in the manuscript.

Field-specific reporting

Please select the one below that is the best fit for your research. If you are not sure, read the appropriate sections before making your selection.

☐ Life sciences ☐ Behavioural & social sciences ☒ Ecological, evolutionary & environmental sciences

For a reference copy of the document with all sections, see nature.com/documents/nr-reporting-summary-flat.pdf

Ecological, evolutionary & environmental sciences study design

All studies must disclose on these points even when the disclosure is negative.

Study description	Genetic loci putatively associated with local adaptation were determined based on their relationship to environmental variation and differentiation among bank vole populations in Britain, and their function and importance in reversing population maladaptation under global warming was assessed based on future climate scenarios. Candidate climate-adaptive loci were identified by partial redundancy analysis (pRDA) following the approach of Capblancq & Forester (2021), which identifies linear combinations of the explanatory variables (four climate variables from WorldClim) that maximize the variance explained in linear combinations of the response variables (multilocus genotypes at 241,099 SNPs loci for 111 individuals from 12 localities), with linear effects of climate variables on genotypes adjusted for population structure by accounting for covariates (individual admixture coefficients).
Research sample	The samples used were tissue samples of bank voles (Clethrionomys glareolus) preserved in ethanol from the authors' collection, selected to represent geographic variation in Britain.
Sampling strategy	The sample size was determined according to the available samples, aiming for a number of 10 individuals per population to ensure a balance between the number of individuals and localities.
Data collection	DNA was extracted at the Institute of Animal Physiology and Genetics of the CAS, and genomic libraries were prepared at the University of Oklahoma by the authors and submitted to the Oklahoma Medical Research Foundation DNA sequencing facility for sequencing.
Timing and spatial scale	Sequence data were obtained on July 11, 2020. Additional data for two individuals that did not provide sufficient data in the first run were obtained on September 12, 2020. Sequence data were obtained from available bank vole samples from previous studies collected largely between 2009 and 2011 at 12 localities in Britain, spanning a distance of 850 km.

Data exclusions	Indels were excluded from called variants, and SNPs were filtered to remove loci with low coverage and quality and a high proportion of missing genotypes.
Reproducibility	Three different approaches were used to detect adaptive loci, and only loci detected by all three methods were subjected to further analysis and interpretation.
Randomization	Because we were concerned with the adaptation of local populations, random assignment of samples to groups was not relevant in our study.
Blinding	Blinding practice was not relevant to our study because no trials were performed.

Did the study involve field work? ☐ Yes ☒ No

Reporting for specific materials, systems and methods

We require information from authors about some types of materials, experimental systems and methods used in many studies. Here, indicate whether each material, system or method listed is relevant to your study. If you are not sure if a list item applies to your research, read the appropriate section before selecting a response.

Materials & experimental systems

n/a	Involved in the study	
☒	☐	Antibodies
☒	☐	Eukaryotic cell lines
☒	☐	Palaeontology and archaeology
☒	☐	Animals and other organisms
☒	☐	Clinical data
☒	☐	Dual use research of concern
☒	☐	Plants

Methods

n/a	Involved in the study	
☒	☐	ChIP-seq
☒	☐	Flow cytometry
☒	☐	MRI-based neuroimaging
